# Machine-Learning-Based Detection of Craving for Gaming Using Multimodal Physiological Signals: Validation of Test-Retest Reliability for Practical Use

**DOI:** 10.3390/s19163475

**Published:** 2019-08-09

**Authors:** Hodam Kim, Laehyun Kim, Chang-Hwan Im

**Affiliations:** 1 Department of Biomedical Engineering, Hanyang University, Seoul 04763, Korea; 2 Center for Bionics, Korea Institute of Science and Technology, Seoul 02792, Korea

**Keywords:** internet gaming disorder, craving for gaming, machine learning, biosignal analysis, test-retest reliability

## Abstract

Internet gaming disorder in adolescents and young adults has become an increasing public concern because of its high prevalence rate and potential risk of alteration of brain functions and organizations. Cue exposure therapy is designed for reducing or maintaining craving, a core factor of relapse of addiction, and is extensively employed in addiction treatment. In a previous study, we proposed a machine-learning-based method to detect craving for gaming using multimodal physiological signals including photoplethysmogram, galvanic skin response, and electrooculogram. Our previous study demonstrated that a craving for gaming could be detected with a fairly high accuracy; however, as the feature vectors for the machine-learning-based detection of the craving of a user were selected based on the physiological data of the user that were recorded on the same day, the effectiveness of the reuse of the machine learning model constructed during the previous experiments, without any further calibration sessions, was still questionable. This “high test-retest reliability” characteristic is of importance for the practical use of the craving detection system because the system needs to be repeatedly applied to the treatment processes as a tool to monitor the efficacy of the treatment. We presented short video clips of three addictive games to nine participants, during which various physiological signals were recorded. This experiment was repeated with different video clips on three different days. Initially, we investigated the test-retest reliability of 14 features used in a craving detection system by computing the intraclass correlation coefficient. Then, we classified whether each participant experienced a craving for gaming in the third experiment using various classifiers—the support vector machine, k-nearest neighbors (kNN), centroid displacement-based kNN, linear discriminant analysis, and random forest—trained with the physiological signals recorded during the first or second experiment. Consequently, the craving/non-craving states in the third experiment were classified with an accuracy that was comparable to that achieved using the data of the same day; thus, demonstrating a high test-retest reliability and the practicality of our craving detection method. In addition, the classification performance was further enhanced by using both datasets of the first and second experiments to train the classifiers, suggesting that an individually customized game craving detection system with high accuracy can be implemented by accumulating datasets recorded on different days under different experimental conditions.

## 1. Introduction

Internet gaming disorder (IGD) was recently listed in the research index of the Diagnostic and Statistical Manual of Mental Disorder [1,2]. IGD in adolescents and young adults is becoming a public concern because of its high prevalence rate and its potential for causing alterations in brain functions and organizations during the brain developmental process [3,4].

Craving is known as a core factor closely associated with both trigger and continuation of addiction [5,6]. Several studies investigated changes in neurophysiological responses during the craving state. For example, Lu et al. reported an increment in the blood volume and respiratory rate while adolescents and young adults with high-risk internet addiction were using the internet [7]. Chang et al. reported a reduced heart rate variability and increased breathing rate during gaming in male college students with a gaming addiction [8]. In our previous study [9], we also investigated changes in various physiological signals, including photoplethysmogram (PPG), galvanic skin response (GSR), and electrooculogram (EOG), in response to the changes in craving for gaming. Short video clips of addictive games were presented to the adolescents with IGD under a virtual reality (VR) environment and the subjective ratings of the current craving for gaming of each participant were collected. Similar to other studies, distinct reductions in the standard deviation of the heart rate and the number of eye blinks, and a significant increase in the mean respiratory rate were observed when a craving for gaming was stimulated. In the same study, we also developed a method to detect the craving for gaming of an individual user with a fairly high accuracy using the multimodal physiological signals (PPG, GSR, and EOG). A machine learning model based on support vector machine (SVM) was used to classify the craving/non-craving states of each individual user.

Recently, several studies have focused on the potential of computer-assisted treatment strategies in addiction therapy as they can potentially improve the accessibility of the patients to the addiction therapy and reduce treatment costs [10,11,12]. Specifically, cue exposure therapy based on the repeated exposure to addiction-related cues was studied as a promising tool to reduce or manage craving [13,14,15,16]. It is expected that the physiological-signal-based craving detection method that we developed previously [9] might be effectively incorporated with various strategies for addiction treatment, as it can quantitatively and continuously monitor the current degree of craving of each individual user. However, our craving detection method demonstrated a limitation, as a relatively long and tedious training session is required prior to the main experimental session to build an optimal SVM model resulting in the highest classification accuracy. Thus, the feature vectors for the machine-learning-based detection of the craving should be selected based on the physiological signals of the user that are recorded on the same day; consequently, degrading the practicality of the developed craving detection method. Indeed, the conventional addiction treatment methods, such as motivational enhancement therapy and cognitive-behavioral therapy, generally require repeated enrollment of a patient for several weeks [11]. Therefore, to create a more practical craving detection system, it should be possible to repeatedly reuse a machine learning model built on the first experimental day in the subsequent series of experiments.

In this study, we investigated whether the machine learning model constructed during the previous experiments could be reused without any further calibration sessions despite the large inter-session variability of physiological signals. We presented short video clips of three addictive games to nine participants, during which various physiological signals, including PPG, GSR, and EOG, were recorded. This experiment was repeated with different video clips on three different days. Initially, we investigated the test-retest reliability of the physiological-signal-based features using an intraclass correlation coefficient (ICC). Subsequently, we identified the craving state of each participant in the third experiment using various classifiers that were trained with features extracted from the previously recorded physiological signals for demonstrating the test-retest reliability characteristic and the practicality of the physiological-signal-based craving detection.

## 2. Methods

### 2.1. Experiment Paradigm

**Figure 1:**
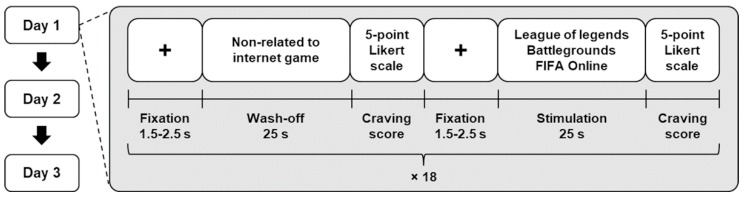
Schematic diagram of the experimental paradigms. We alternately presented video clips that were irrelevant (mostly natural scenery) and relevant (FIFA Online, League of Legends, and Battlegrounds) to online games. A total of 36 videos were presented to each participant, all of which were different from each other and counterbalanced.

Eighteen short video clips that were not associated with games (referred to as the wash-off trials) and eighteen video clips of addictive games (referred to as the stimulation trials) were alternately presented to the participants using a commercial head-mounted display device (HTC VIVE^TM^ VR system; HTC Co., Ltd., Xindian District, New Taipei City, Taiwan). Video clips of three addictive games (League of Legends^TM^, Battlegrounds^TM^, and FIFA Online^TM^), which were three best-selling online games in South Korea at the time of the experiments, were randomly presented to the participants in the stimulation trials; moreover, the number of appearances of each game was set to be identical (six times for each game). Each video clip was 25 s long. Immediately after the presentation of each video clip, a self-reported questionnaire using a 5-point Likert scale for rating the subjective degree of craving for gaming was presented to the participants. The questionnaire consisted of the statement: “Please select a number (1–5) that best describes your current craving for gaming,” and the five scale points were labeled according to the degree of craving for gaming (1 = I do not feel any craving for gaming, 3 = I feel craving for gaming, 5 = I feel a very strong craving for gaming). This experimental procedure was repeated thrice over three different days (hereafter referred to as “Day 1”, “Day 2”, and “Day 3”); further, the video clips used for each experiment were prepared differently. Figure 1 illustrates a schematic diagram of the overall experimental paradigm.

### 2.2. Participants

A total of nine male college students (Age: 20.60 ± 1.14, denoted as S1, S2, …, S9) participated in our experiments. An expert psychiatrist, who had been studying this research topic for more than two years, screened the participants and applied the Young’s Internet Addiction test [17,18] to evaluate the severity of their addiction to gaming (Young’s scales of nine subjects: 62.44 ± 10.10). Table 1 lists the demographic details of all participants. All participants had normal or corrected-to-normal vision. The participants did not have a history of neurological, psychiatric, or other severe diseases that could affect the experimental results. Each participant was verbally informed of the detailed experimental protocol and signed a consent form prior to the experiment. Monetary reimbursement was provided to each participant after the completion of the three repeated experiments performed on different days. The start times of the three repeated experiments were set identically for each participant (e.g., 3 p.m. for participant S1, 4 p.m. for participant S2, etc.). In addition, other variables that might affect the physiological signals were controlled. For example, the participants were instructed not to drink alcoholic beverages or perform strenuous activities 24 h prior to the experiments to eliminate fatigue-related factors. Moreover, the smokers amongst the participants were instructed not to smoke for 2 h before the experiment.

### 2.3. Acquisition of Multimodal Biosignals

A commercial biosignal recording system (ActiveTwo; BioSemi, Amsterdam, The Netherlands) was used to record PPG, GSR, and EOG signals. Similar to the experiment in our previous study [9], the PPG and GSR signals were recorded from the left hand (PPG: left index finger; GSR: left middle and ring fingers). Further, the EOG signals were recorded from four locations around the eyes; the locations were above and below the right eye and the outer sides of both eyes. The reference and ground electrodes were attached to the right and left mastoids, respectively; moreover, the sampling frequency of the recorded signals was set at 2048 Hz.

### 2.4. Feature Extraction

For each 25 s trial, the physiological signals were pre-processed and 14 feature candidates were extracted from the recorded PPG, GSR, and EOG signals [9]. Each physiological signal was preprocessed using the procedures discussed subsequently. The PPG signal was filtered using a 5th-order median filter and a 4th-order Butterworth bandpass filter with cutoff frequencies of 0.1 Hz and 10 Hz. The GSR signals were down-sampled from 2048 Hz to 16 Hz and then lowpass-filtered with a 0.2 Hz cutoff frequency. In addition, a linear detrending process was applied to the filtered GSR signals. The four EOG signals were down-sampled from 2048 Hz to 16 Hz. The vertical EOG component was acquired by calculating the difference between the signals recorded above and below the right eye; further, the horizontal EOG component was derived by calculating the difference between the signals recorded from the outer side of both eyes (right–left). Two EOG components were median-filtered with a window size of seven points, and the baselines of the two components were drifted by subtracting the median value of each component [19].

We tracked the changes in the heart rate (HR) and respiratory rate (RR) at intervals between 10 s and 25 s of each trial by applying the adaptive infinite impulse response filter-based RR estimator (please refer to [20] for more details). Then the mean and standard deviation of HR and RR (mHR, stdHR, mRR, and stdRR) were evaluated from the estimated HR and RR values. The preprocessed GSR signal was normalized by z-score transformation, and the peaks from the normalized GSR signal were identified by the zero-crossing method. The mean amplitude of normalized skin conductance (mNSC) and minimum amplitude of normalized skin conductance (minNSC) can then be easily evaluated. Eye blinking could be detected from the vertical EOG component using an automated eye-blink detection algorithm [21]. The number of eye blinks (NE) detected from the vertical EOG component was counted, and the detected eye blinking intervals from the vertical EOG were removed and linearly interpolated. The horizontal and vertical saccadic eye movements were estimated from two EOG components using the continuous wavelet transform-saccade detection algorithm [22], and the four feature candidates corresponding to the degree of saccadic eye movements (degree of horizontal saccadic movement (DHSM), degree of vertical saccadic movement (DVSM), mean of DHSM and DVSM (mDHV), and degree of saccadic movement (DSM)) were evaluated. Finally, the values of covariance (covariance of horizontal EOG and vertical EOG (CHV), covariance of horizontal EOG and PPG (CHP), and covariance of vertical EOG and PPG (CVP)) among the vertical and horizontal EOG components and PPG values were calculated (please refer to the Appendix in [9] for more details on the evaluation of these features).

### 2.5. Classification of Craving States and Feature Selection

In our previous study [9], the self-reported craving scores of 47 participants after watching gameplay videos were significantly higher than those after watching wash-off videos. The stimulation and wash-off trials were labeled as high- and low-craving states, respectively. We classified the two craving states using the SVM and evaluated the classification accuracy using a 10-fold cross validation individually for each participant. In this study, similar to our previous study, the two types of trials were labeled as high- and low-craving states. We then classified the binary craving states of each participant from the recorded physiological signals using SVM (open software package LIBSVM [23]; kernel: radial basis kernel; cost: 1; gamma: 1/the number of features). We evaluated the classification accuracy of binary craving states in each trial of the “Day 3” experiment assuming the four different subsequently described conditions. First, the classification accuracy was evaluated using only the “Day 3” experimental data based on the 6-fold cross validation technique (30 trials were used as training data and six trials were used as testing data in each fold), when different feature vectors are selected for each fold and each participant. This condition was identical to the conventional approach adopted in our previous study [9], which is not practical considering that a user is required to participate in a 15 min long training session every time the user desires to use the craving detection system. Second, all trials of the “Day 1” experiment were used to train the model of classifiers. Third, all trials of the “Day 2” experiment were used as the training data. Fourth, all trials of the “Day 1” and “Day 2” experiments were used as the training data to build the model of classifiers. We used the Fisher score method [24] to select an optimal set of features and to reduce the potential risk of overfitting. We selected two features with the highest Fisher scores among the 14 feature candidates for unbiased comparison and then evaluated the classification accuracy using the classifier trained with the selected features. We limited the number of features to two, considering the small training sample size. Note that different feature vectors were used for each condition and each participant. Additionally, we classified the binary craving states using other classifiers, i.e., k-nearest neighbors (kNN) [25], centroid displacement-based kNN (CDNN) [26], linear discriminant analysis (LDA) [24], and random forest [27], in addition to the SVM. We chose the number of nearest neighbors in the kNN algorithm to be 17 and in the CDNN algorithm to be the number of training data in our experiments. Further, we selected a feature with the highest Fisher score to train the four classifiers.

### 2.6. Statistical Analysis

Since the number of participants was limited, non-parametric statistical testing was applied. For the assessment of the difference between two sets, the Wilcoxon signed rank test or the Wilcoxon rank sum test was selectively used depending on whether the two sets were paired or not. The Friedman test was used when the differences among three or more sets were tested; further, the Bonferroni correction was applied for the multiple comparison corrections in the post-hoc analyses. In addition, to estimate the test-retest reliability of the 14 features, the ICC was calculated using a single-measurement, absolute agreement, two-way mixed-effects model [28,29,30]. The ICC value of each feature for each trial was calculated using the mean value of the feature at the trial.

## 3. Results

### 3.1. Self-Reported Craving Score

We first confirmed whether the craving for gaming was effectively stimulated through statistical analysis of the craving scores rated by each subject. Table 1 lists the median values and interquartile ranges of the craving scores for wash-off and stimulation trials. The difference between the craving scores for two types of trials was tested using the Wilcoxon rank sum test for each participant. In all cases, the craving scores obtained after the stimulation trials were significantly higher than those obtained after the wash-off trials (*p* < 0.003), implying that the gaming videos used in our experiments could effectively stimulate the cravings for gaming among the participants.

### 3.2. Test-Retest Reliability of Features

Table 2 lists the ICC values of 14 feature candidates. ICC values of two features (stdHR, mHR) based on PPG signal, ICC values of six features (NE, DHSM, DVSM, mDHV, DSM, CHV) based on EOG signal, and ICC values of CHP based on both EOG and PPG signals were statistically significant in both wash-off and stimulation trials (*p* < 0.05). The existence of features with high ICC suggests the possibility of using a classifier built using datasets recorded in other experimental sessions for the classification of the current craving state. On the other hand, it was observed that features based on RR (stdRR and mRR) and GSR (mNSC and minNSC) showed relatively low ICC values, suggesting that the RR- and GSR-based features might not be appropriate to be used repeatedly in practical scenarios.

### 3.3. Classification of Craving State

**Figure 2:**
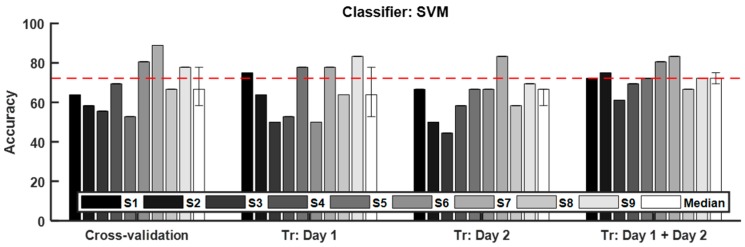
Accuracy in classifying craving states using multimodal biosignals measured during “Day 3” experiments. “Cross-validation” represents the 6-fold cross validation using the “Day 3” data. “Tr: Day 1” implies that the “Day 1” dataset was used as training data. “Tr: Day 2” implies that “Day 2” dataset was used as training data. “Tr: Day 1 + Day 2” implies that both “Day 1” and “Day 2” datasets were simultaneously used as training data. The error bars of median accuracies indicate the first and third quartile of the classification accuracies of all subjects. The red dashed line represents the median classification accuracy for “Tr: Day 1 + Day 2”.

As already described in Section 2.5, we classified the craving states of a participant using physiological signals acquired on “‘Day 3” under four different conditions: (1) 6-fold cross validation (denoted as “cross-validation” in Figure 2), (2) the “Day 1” dataset used as training data (denoted as “Tr: Day 1” in Figure 2), (3) the “Day 2” dataset used as training data (denoted as “Tr: Day 2” in Figure 2), and (4) both “Day 1” and “Day 2” datasets used as training data (denoted as “Tr: Day 1 + Day 2” in Figure 2). The Friedman test and Wilcoxon signed rank test were used to test the statistical significance of difference among the classification accuracies for the four conditions and the difference among the classification accuracies achieved using five classifiers. Table 3 lists the median and the interquartile range of the classification accuracies across subjects obtained using five kinds of classifiers (SVM, kNN, CDNN, LDA, and random forest). Statistical analyses reported that there was no statistically meaningful difference in the median values. Nevertheless, the interquartile range of classification accuracy for SVM trained with both “Day 1” and “Day 2” datasets was the least, implying that the SVM showed the most consistent classification performance among the five classifiers tested in this study. Since this ‘small variability’ characteristic might be important in realizing a more robust craving detection system, the SVM was employed as the classifier model in the further analyses. Figure 2 shows the classification accuracy of each subject and the median classification accuracy across subjects achieved using SVM. The median accuracies for the four conditions were 66.67%, 63.89%, 66.67%, and 72.22%, respectively. The Friedman test showed a marginally significant difference among four conditions (*p* = 0.0567). The median classification accuracies for the second (Tr: Day 1) and third (Tr: Day 2) conditions were comparable with each other; however, the classification accuracy achieved when “‘Day 1” and “Day 2” datasets were used together as training data was significantly higher than those in the second and third conditions (Bonferroni corrected *p* < 0.05). It is worthwhile to note that only the “Tr: Day 1 + Day 2” condition exhibited a median classification accuracy above 70%, which has been generally regarded as a threshold accuracy for practical use of binary classification, especially in the field of brain-computer interfaces [31].

## 4. Discussion

Presenting images or videos related to addictive objects and collecting self-rated craving scores is a widely used process to elicit craving [31,32,33]. In our previous study [9], we presented video clips of online gameplay and natural scenery to the participants with IGD to stimulate and diminish the craving for gaming, respectively. The gameplay videos used in the previous experiments proved to be sufficiently effective in creating a craving for gaming in all participants regardless of the severity of IGD. In the same study, we also demonstrated that the craving for gaming in an individual user could be detected using multimodal physiological signals. In the current study, to overcome the limitation of the machine-learning-based craving detection method that generally requires long and tedious training time, we further investigated the test-retest reliability and practicality of the craving detection method.

In this study, we used a machine learning model built using physiological signals recorded in previous experiments for the classification of craving/non-craving states of young adults with IGD to confirm the test-retest reliability of our physiological-signal-based craving detection method. Our results showed that the craving of the participants for gaming in the third experiment could be classified using the data acquired on a previous day with an accuracy comparable to that achieved using the data acquired on the same day, consequently, demonstrating the high test-retest reliability of this method. In addition, the classification performance could be further enhanced by using combined datasets from the first and second experiments to build the classifier, suggesting that an individually customized craving detection system with high accuracy might be implemented by accumulating datasets recorded under different experimental conditions.

The median classification accuracy for the 6-fold cross validation (first condition) was relatively lower than that reported in our previous study [9]. The primary reason for the lowered classification accuracy might be the restriction in the number of features used for the classification. In our previous study, the number of features totaled 14, as we tested all possible combinations of features instead of selecting a few features; however, in the present study, only two features were selected using the Fisher score method. We restricted the number of features to two because the total number of trials was just 36 in the present experiments; thus, there was a possibility of overfitting when larger numbers of features were used. In addition, we wanted to ensure that the comparison conditions were as unbiased as possible by matching the feature dimensions among the four different conditions. Although the median accuracies achieved in our classifications might not be optimal, the relative comparisons among different conditions were considered to be more important in the present investigation of test-retest reliability. As expected, our results demonstrated that the cravings for gaming could be detected using a machine learning model built with data acquired from previous experiments with an accuracy comparable to that achieved using the data acquired on the same day. Conversely, the accuracy of detecting craving states can be enhanced using a majority voting scheme in the practical scenarios when rapid feedbacks are not required. The majority voting scheme, which selects a specific class that a majority among multiple trials agrees with, has shown a significant increase in classification accuracy for several physiological-signal-based classification problems [34,35,36,37]. This majority voting scheme will be employed in our future studies to implement a real-time craving monitoring system incorporated with addiction treatment strategies.

When both datasets from the first and second experiments were used to build the SVM model, the classification accuracy was considerably enhanced, suggesting that the accumulation of training datasets recorded under different experimental conditions (i.e., several variable factors that might affect the recorded physiological signals, such as different health conditions, different baselines of physiological signals, and use of different gameplay video clips) might help to increase the overall performance of the physiological-signal-based craving detection system. However, these findings cannot be generalized only from our results owing to the limited numbers of participants enrolled in this study. It was relatively difficult to ensure that participants with a high value of Young’s scale enrolled in the three consecutive experiments. In fact, several participants refused to participate in the second experiment after experiencing the first experiment. In addition, data from two participants who participated in all three experiments were excluded from the analyses because they did not feel any craving for gaming on certain days (i.e., there was no significant difference in the self-reported craving scores for the two different types of video clips). In our future study, our results have to be further generalized by enrolling more participants and tracking the changes in the test-retest reliability for a longer period of time. In addition, building a generic classifier model using physiological data of a group of individuals for the classification of an individual’s craving states is a promising topic that we want to pursue in our future studies.

## 5. Conclusions

The primary aim of this study was to investigate whether craving for gaming could be detected using physiological signals recorded on previous days. Therefore, we repeated an experiment to stimulate craving for gaming thrice for each participant, and then observed the test-retest reliability of the 14 features and the physiological-signal-based craving detection method. Our method exhibited high test-retest reliability, suggesting that only a few calibration and training sessions would suffice for the repeated applications of our craving detection method in practical addiction treatment scenarios.

## Figures and Tables

**Table 1:** Demographic and median (interquartile range) of self-reported craving scores of participants.

Subj.	Age	Young Scale	Self-Reported Craving Score								
			Day 1			Day 2			Day 3		
			Wo	S	Sig.	Wo	S	Sig.	Wo	S	Sig.
S1	19	64	1 (1)	4 (1)	**	1 (1)	4.5 (1)	**	1 (1)	4.5 (2)	**
S2	20	62	1 (1)	4 (2)	**	1 (1)	4 (1)	**	1 (0)	3 (2)	**
S3	21	55	2.5 (1)	3 (1)	*	2 (0)	3 (1)	*	2 (0)	3 (1)	**
S4	21	81	2 (1)	4 (2)	**	1 (1)	4 (1)	**	2 (1)	4.5 (1)	**
S5	22	66	1 (1)	3 (1)	**	1.5 (1)	4 (1)	**	1 (0)	3 (1)	**
S6	23	71	2 (1)	3.5 (1)	**	2 (0)	3 (0)	**	2 (1)	3 (0)	**
S7	20	56	2 (1)	4 (0)	**	2 (0)	4 (0)	**	2 (1)	4 (0)	**
S8	21	49	1 (0)	4 (3)	**	2 (1)	4 (1)	**	2 (1)	4 (1)	**
S9	19	53	1.5 (1)	3.5 (1)	**	2 (0)	3 (1)	**	2 (1)	4 (2)	**

Subj.: Subject; Wo: Wash-off trial; S: Stimulation trial; Sig.: Significance; * *p* < 0.003; ** *p* < 0.001.

**Table 2:** Intraclass correlation coefficient (ICC) of 14 features.

Feature Number	Wash-Off Trial		Stimulation Trial	
	ICC	*p*-Value	ICC	*p*-Value
1	0.80	0.00	0.65	0.00
2	0.50	0.01	0.56	0.00
3	0.68	0.00	0.32	0.07
4	0.53	0.01	0.21	0.18
5	0.39	0.05	0.01	0.45
6	0.17	0.20	0.11	0.31
7	0.54	0.01	0.47	0.02
8	0.70	0.00	0.70	0.00
9	0.50	0.02	0.58	0.01
10	0.70	0.00	0.72	0.00
11	0.70	0.00	0.72	0.00
12	0.62	0.00	0.56	0.01
13	0.46	0.01	0.61	0.00
14	0.51	0.02	0.14	0.26

Feature number: 1 = stdHR; 2 = mHR; 3 = stdRR; 4 = mRR; 5 = mNSC; 6 = minNSC; 7 = NE; 8 = DHSM; 9 = DVSM; 10 = mDHV; 11 = DSM; 12 = CHV; 13 = CHP; 14 = CVP.

**Table 3:** Median and interquartile range of classification accuracies using various classifiers.

Classifier	Median (Interquartile Range) of Classification Accuracy (%)			
	Cross-Validation	Tr: Day 1	Tr: Day 2	Tr: Day 1 + Day 2
SVM	66.67 (20.83)	63.89 (25.69)	66.67 (11.11)	72.22 (7.64)
LDA	75.00 (17.36)	63.89 (31.25)	61.11 (23.61)	72.22 (15.97)
kNN	69.44 (20.14)	58.33 (19.44)	69.44 (20.83)	69.44 (13.89))
CDNN	75.00 (17.36)	63.89 (31.25)	61.11 (23.61)	72.22 (15.97)
Random forest	66.67 (14.58)	55.56 (17.36)	58.33 (11.81)	72.22 (12.50)
